# Influence of Interleukin-4 and Interleukin-13 on serum immunoglobulin E in house dust mite allergy

**DOI:** 10.1186/2045-7022-4-S2-P22

**Published:** 2014-03-17

**Authors:** Carmen Panaitescu, Laura Marusciac, Luminita Cernescu

**Affiliations:** 1 University of Medicine and Pharmacy Victor Babes Timisoara, Department of Functional Sciences, Timisoara, Romania

## Background

Th2 cells are the central mediators of the allergic immune response, as their activation induces it by cytokine release. The key cytokines expressed by Th2 cells and involved in the pathogenesis of allergic airway inflammation and immunoglobulin E (IgE) production are interleukin-4 (IL-4), IL-5, IL-10 and IL-13. The aim of study was to evaluate the in vitro effect of Der p 1, the major allergen of Dermatophagoides pteronyssinus (house dust mite, HDM), on IL-4 and IL-13 production by naive CD4+ T cells cocultured with dendritic cells (DCs) from HDM allergic patients, as well as their influence on serum IgE levels.

## Method

The study groups consisted of 9 patients allergic to HDM, diagnosed with allergic rhinitis ± allergic asthma, and 7 healthy controls. Skin prick tests (SPT) to HDM extract were performed in all patients. Serum CD14+ monocytes were isolated from venous peripheral blood of the HDM allergic patients and differentiated into immature DCs using GM-CSF and IL-4. The generated DCs were pulsed for 24 hours with Der p 1, and then cocultured with autologous naive CD4+ T cells for 24 hours. Supernatant removed after each phase was assayed for IL-4, IL-13 and IFN-γ levels. Total and specific serum IgE levels were assayed by ELISA.

## Results

HDM allergic patients were characterized by a highly positive SPT for Dermatophagoides pteronyssinus antigens, total serum IgE levels between 133.4 and 3779 IU/mL (1112.76±1114.17 IU/mL), and Der p 1-specific IgE of 22.98±13.02 IU/mL, while in healthy donors the SPT was negative, and total serum IgE was 66.98±53.49 IU/mL. IL-4 serum levels were extremely significantly higher in allergic patients (0.038±0.023 pg/mL) in comparison to healthy donors (0.008±0.006 pg/mL) (p<0.001). In allergic patients, autologous T cells cocultured with Der p 1-pulsed DCs produced higher levels of IL-4 (5.576±8.061 pg/mL) and lower amounts of IFN-γ (72.116±180.559 pg/mL) than healthy donors. A significant correlation was observed between IL-4 production and Der p-specific IgE levels, and between IL-13 production by Der p 1-pulsed DCs and total IgE levels.

## Conclusion

Both IL-4 and IL-13 are involved in the development of an allergic immune response in HDM allergic patients, but their importance in the inflammatory process differs. The data suggests that IL-4 is more important than IL-13 for specific IgE production, whereas IL-13 seems to be the main cytokine that stimulates total IgE production.

